# The Profession of British Nurses: I. Plain Words on Registration

**Published:** 1917-03-31

**Authors:** 


### THE MATRONS' AND SISTERS' DEPARTMENT.

### THE PROFESSION OF BRITISH NURSES.

### I. Plain Words on Registration.

SCIENTIFIC ANALYSIS.

For many years the nursing world has been perturbed concerning State Registration, and during recent months the air has been ringing with such party cries as "Registrationists" and "Anti-Registrationists"—expressions which are not alone misleading, but, to the rank and file of the profession, embarrassing. In their interests it is desirable thoroughly to examine the whole subject so that there may remain no room for ambiguity or doubt. Happily it is possible to make such an examination without acrimony or bias, just as it is possible to analyse water or air. Nobody disputes their composition. The chemist can measure them, weigh them, divide them into their component parts—and there is no argument, no recrimination, no bitterness. It has, unfortunately, become common in modern journalism to confound principles with persons, and oftentimes, in cases where a writer finds himself confronted with a statement which conflicts with his own views, to combat the statement by a virulent personal attack on its author. Here is a case in point taken from the *British Journal of Nursing* of March 24:—

The following paragraph has been sent for publication by Miss Mundy, Hon. Sec. Manchester Branch, N.U.T.N., from the Royal Infirmary, Manchester:—"The following resolution was sent out to 355 members, the result being 145 not returned, 202 in the affirmative, 5 in the negative, 3 too late:—*Resolved*: 'That the Manchester Branch very much regrets the action of the Council in opposing the College of Nursing, in its endeavours to obtain union, and begs the Council of the N.U.T.N. to change its policy, and if possible to co-operate with the College.'"

We presume this entirely misleading resolution has been drafted by Miss Sparshott, the chairman of the Manchester Branch, and known throughout the nursing profession for years as one of the most intolerant opposers of the State Registration of Nurses, and who recently expressed the opinion that it was the place of matrons "to suggest, direct, and control the College of Nursing"!

Argument of this description fails to convince.

#### Ideals True and False.

Not everybody appreciates the importance of clearly understanding whether movements are of major or only of minor importance, whether they are wholly or only partially beneficial. Nevertheless such clear understanding is extremely important. Man's mental and moral attitude determines his life's conduct. The difference between a Christian gentleman and a barbarian is due to their diverse conceptions of the Deity. The present conflict in Europe when analysed and examined resolves itself into a conflict of ideals. It may seem a platitude to say that ignorance is the main cause of error, but all the same it is by no means generally recognised that it is so. Scores, nay hundreds and probably thousands of high-minded and clever women have spent priceless years battling for Registration, believing it to be something that it is not, and tens of thousands have blindly followed their leaders without pausing to inquire whither they were bound, or what treasure was buried there. Have you ever observed a flock of sheep walking along the highway? If one sheep jumps over a real or imaginary obstacle every other sheep that follows in his wake also jumps. This may be observed even in the streets of a crowded city, and is attributed to some far-off instinct derived from ancestors whose home was in the mountains, and who followed thus their leader when flying from an enemy in pursuit. Nurses are no more like unto sheep than the rest of humanity. The follow-my-leader instinct pervades every section of the race. In these circumstances it will probably shock many and appear a dangerous heresy to suggest that State Registration is for nurses very largely a Will-o'-the-wisp, a false beacon luring them to a phantom promised land. Nature often provides these *ignes fatui* for unwary, if weary, wayfarers. The mirage in the desert is a vision of a tranquil lake surrounded by shady trees in regions parched and bare. Shipwrecked mariners sometimes see on the horizon images of mountains or of gallant ships riding with sails unfurled, when in reality there is nothing but a waste of waters. State Registration for nurses has indeed been a veritable Will-o-the-wisp, with this difference that, immediately or ultimately it will become an accomplished fact. The goal will be reached.

Lest peradventure we may be falsely branded as enemies of State Registration, let us hasten to express the earnest hope that this may take place immediately rather than ultimately. The sooner the pursuit is ended, the sooner shall we be free to follow after genuine reforms. State Registration holds for nurses its limited uses, and these will be duly set forth in their proper proportion as this analysis proceeds. What the nurse most urgently needs is material benefit; some tangible good which she fondly believes is wrapt up in State Registration. All this bandying of words, these party shibboleths, are barren save possibly of transient glory for the leaders of faction. The great majority of hardworked, earnest women whose lives are devoted to the cause of suffering humanity, who enjoy very few of the sweets, will derive far less benefit from State Registration than they have been led to believe, and it is of untold importance to them that they should realise the truth. Like the Christian and the barbarian; like the British soldier and the Hun, the choice of a true or false ideal will determine the nurse's destiny. Her close attention, therefore, to a thorough examination of Registration and all that it means becomes for her a serious duty.

